# Sex differences for clinical and pathological correlates of APOEe4 in people with Lewy body pathology

**DOI:** 10.1002/alz.092039

**Published:** 2025-01-03

**Authors:** Ece Bayram, Irene Litvan

**Affiliations:** ^1^ University of California San Diego, La Jolla, CA USA

## Abstract

**Background:**

In people with Lewy body (LB) pathology, Alzheimer’s (AD) co‐pathology staging has stronger association with cognition for females, and LB pathology staging has stronger association with behavioral and motor symptoms for males. As APOEe4 allele is a risk factor for LB and AD pathologies, in both females and males; we investigated whether APOEe4 association with clinical profile and pathology staging differs by sex in people with LB pathology.

**Methods:**

Data were obtained from the National Alzheimer’s Coordinating Center Uniform Data Set (UDS), Neuropathology and Genetic Data Set for UDS visits conducted between September 2005 and August 2019. 294 females and 490 males with available APOEe4 count data from 33 Alzheimer’s Disease Research Centers, with brainstem, limbic or neocortical LB pathology were included, excluding those with other neuropathologic diagnoses. Interactions between sex and APOE ε4 count for pathology staging (LB, AD), clinical diagnosis (LB disease, AD), dementia likelihood and severity (CDR® Dementia Staging Instrument‐Sum of Boxes), behavioral symptom severity (Neuropsychiatric Inventory Questionnaire) at last visit were assessed with linear models controlling for age.

**Results:**

40.5% of females, 40.8% of males had one APOEe4; 12.2% of females, 12.9% of males had two APOEe4 alleles (*p* = .95). Compared to males, females were older at last visit and died older. APOEe4 was not associated with LB pathology staging. Compared to males, APOEe4 was associated with higher level of AD pathology staging (B = 1.48 for females, B = 0.66 for males; *p*<.001); higher likelihood of AD clinical diagnosis (B = 1.30 for females, B = 0.49 for males; *p*<.001); lower likelihood of LB disease clinical diagnosis (B = ‐0.76 for females, B = ‐0.16, *p*<.001); higher likelihood for dementia (B = 1.76 for females, B = 1.21 for males; *p*<.001) and more severe dementia (B = 2.69 for females, B = 1.53 for males; *p*<.001) for females. Compared to females, APOEe4 was associated with more severe behavioral symptoms for males (B = 0.36 for females, B = 1.34 for males; *p*<.001).

**Conclusions:**

In people with LB pathology, APOEe4 is associated with AD co‐pathology staging, clinical diagnosis, dementia likelihood, dementia and behavioral symptom severity. However, these associations can be different for females and males. Clinical and pathological associations of genetic factors can differ by sex in LB disease.

